# Optimal Scaling of Critical Size for Metamorphosis in the Genus *Drosophila*

**DOI:** 10.1016/j.isci.2019.09.033

**Published:** 2019-09-27

**Authors:** Ken-ichi Hironaka, Koichi Fujimoto, Takashi Nishimura

**Affiliations:** 1 Laboratory of Theoretical Biology, Department of Biological Sciences, Osaka University, Osaka 560-0043, Japan; 2 Laboratory for Growth Control Signaling, RIKEN Center for Biosystems Dynamics Research (BDR), Hyogo 650-0047, Japan

**Keywords:** Biological Sciences, Developmental Biology, Evolutionary Developmental Biology, Mathematical Modelling

## Abstract

Juveniles must reach a critical body size to become a mature adult. Molecular determinants of critical size have been studied, but the evolutionary importance of critical size is still unclear. Here, using nine fly species, we show that interspecific variation in organism size can be explained solely by species-specific critical size. The observed variation in critical size quantitatively agrees with the interspecific scaling relationship predicted by the life history model, which hypothesizes that critical size mediates an energy allocation switch between juvenile and adult tissues. The mechanism underlying critical size scaling is explained by an inverse relationship between growth duration and growth rate, which cancels out their contributions to the final size. Finally, we show that evolutionary changes in growth duration can be traced back to the scaling of ecdysteroid hormone dynamics. We conclude that critical size adaptively optimizes energy allocation, and has a central role in organism size determination.

## Highlights

• We quantified the growth parameters including critical size in nine fly species
• The optimal scaling of critical size predicted by a life history model is verified
• Scaling is explained by an inverse relation between growth duration and growth rate
• Species evolution of growth duration can be traced back to that of hormone dynamics

## Graphical Abstract

**Figure:**
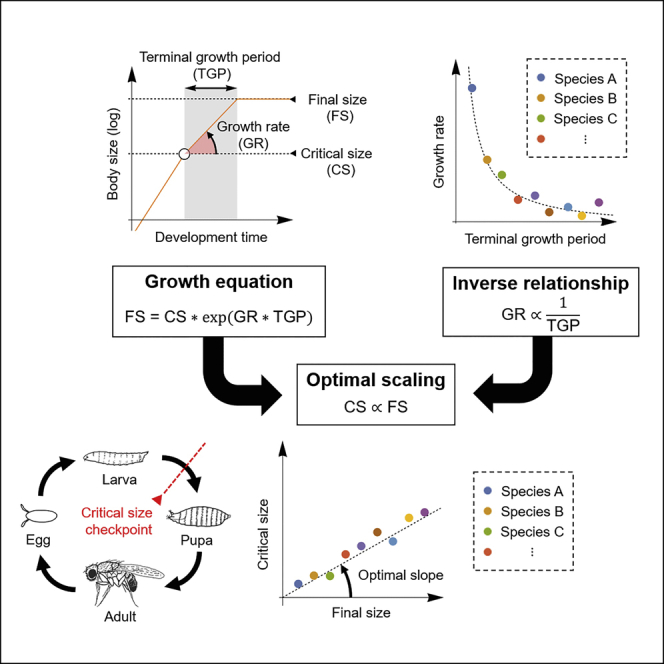

## Introduction

**Figure 1:**
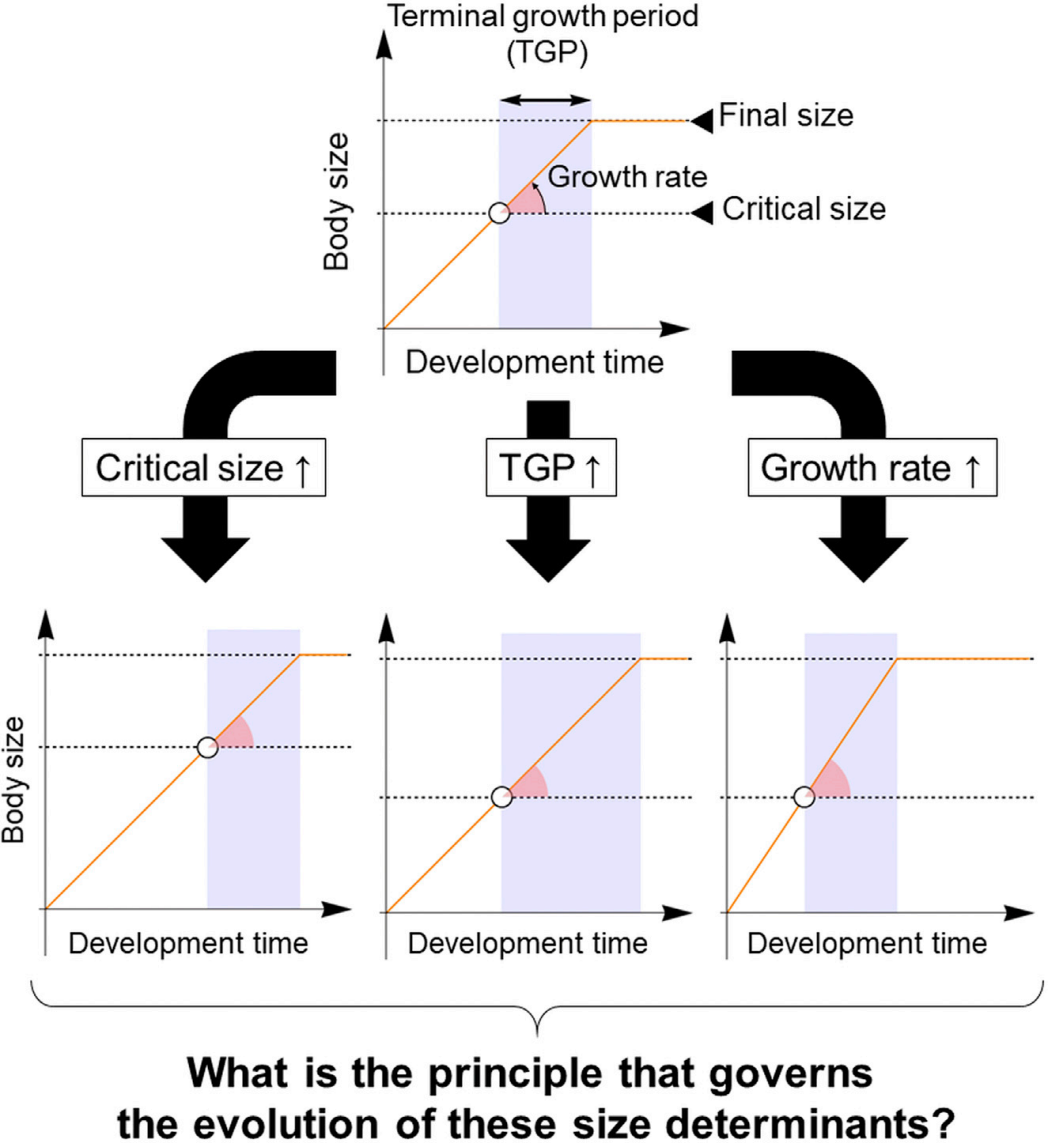
Evolutionary Questions Regarding Developmental Thresholds In many organisms, adult maturation is triggered by reaching a critical size, also called a developmental threshold. Because there is a lag time between reaching critical size (circle) and growth cessation, the critical size is usually much smaller than the final size. Thus, final body size can be delineated by three factors: critical size (left panel), duration of the terminal growth period (TGP, center panel), and growth rate during the TGP (right panel). Potentially, any of these three determinants can contribute to body size evolution.

Body size is a common and important trait that affects the physiological and ecological performance of an organism (McMahon and Bonner, 1983, Schmidt-Nielsen, 1984, Thompson, 1942). Although molecular genetics studies have clarified mechanisms controlling organ growth, the mechanisms that determine organism size remain largely unknown because organism growth and organ growth are coordinated but distinct phenomena (Hariharan, 2015, Pan, 2007, Tumaneng et al., 2012). Moreover, given that many animals do not grow indeterminately even with sufficient food, not only growth but also growth cessation is important in determining final size (Boulan et al., 2015, Cook and Tyers, 2007, Edgar, 2006, Gokhale and Shingleton, 2015, Stern, 2003). At the organism level, growth cessation often accompanies a life history transition such as sexual maturation or metamorphosis, which are mediated by the action of hormones such as ecdysteroid hormone (insects), thyroid hormone (amphibians), and sex steroid hormones (mammals). These hormones are usually synthesized only after juveniles reach a certain body size, called the “developmental threshold” or “critical size” (Day and Rowe, 2002). Therefore, the timing of growth cessation is pre-determined upon attainment of critical size, which occurs much earlier than growth cessation (Figure 1). For example, humans enter puberty when reaching a critical body weight and then complete developmental growth and sexual maturation during puberty by the action of sex steroid hormones (Frisch and Revelle, 1971, Frisch and Revelle, 1970). Eventually, final body size can be delineated by three factors: critical size, terminal growth period (TGP) between critical size attainment and growth cessation, and growth rate during the TGP (Figure 1).

Previous studies have reported that critical size, TGP, or growth rate can contribute to body size differences at the intraspecific and interstrain level (D’Amico et al., 2001, De Moed et al., 1999, Nijhout et al., 2006, Partridge et al., 1999). This finding presents the question of whether organisms can freely choose between these three size determinants to attain a desirable body size over the course of evolution or if there are principles that govern the evolution of size determinants. To address this issue, it is important to understand the role of critical size in evolution and development. However, critical size has so far been defined directly as a priori physiological constraints without any clear connection to fitness, making it difficult to infer the role of critical size (Day and Rowe, 2002).

Recent studies of holometabolous insects such as *Drosophila* and *Manduca* have provided some insights into the adaptive significance of critical size (Edgar, 2006, Nijhout et al., 2014, Riddiford, 2011). During the larval stage, individuals must reach a critical size to initiate metamorphosis. Attaining critical size induces the prothoracic gland to secrete ecdysteroid hormone, which triggers metamorphic events such as growth cessation and pupation (Koyama et al., 2014, Mirth et al., 2005, Ohhara et al., 2017). In addition, imaginal tissues (i.e., precursors of adult organs) begin rapid growth after attaining critical size in an ecdysteroid-dependent manner (Bryant and Levinson, 1985, Mirth et al., 2009, Truman et al., 2006). Based on these observations, we hypothesized that critical size mediates an energy allocation switch that slows down the growth of larval tissues and speeds up the growth of adult imaginal tissues (Hironaka and Morishita, 2017). Our model links critical size with fitness and predicts an evolutionary principle for size determination, based on an interspecific scaling relationship between critical size and final larval size (Figure 1C). This scaling relationship is quantitatively testable because the theoretical scaling coefficient is determined solely by two parameters: growth scaling exponent and energy reallocation efficiency.

Here, to test “energy allocation switch” hypothesis of critical size, we examined the interspecific variation in critical size and other growth characteristics using nine *Drosophila* species. Our data confirm that the optimal scaling relationship of critical size holds across the genus *Drosophila*, providing a strong evidence for the energy allocation switch. The mechanism underlying critical size scaling can be explained by an inverse relationship between the growth duration and the growth rate, which cancels out their effects on the final size and makes critical size contribute more directly to the final size. Finally, we show that evolutionary change of the growth duration, or heterochrony, can be traced back to the temporal scaling of ecdysteroid hormone dynamics. Our results suggest that critical size is evolved to optimize energy allocation between different organs, which provides a previously unrecognized principle of organism size evolution.

## Results and Discussion

### The Scaling of Critical Size Is Derived from an Optimal Life History Model for Holometabolous Insects

**Figure 2:**
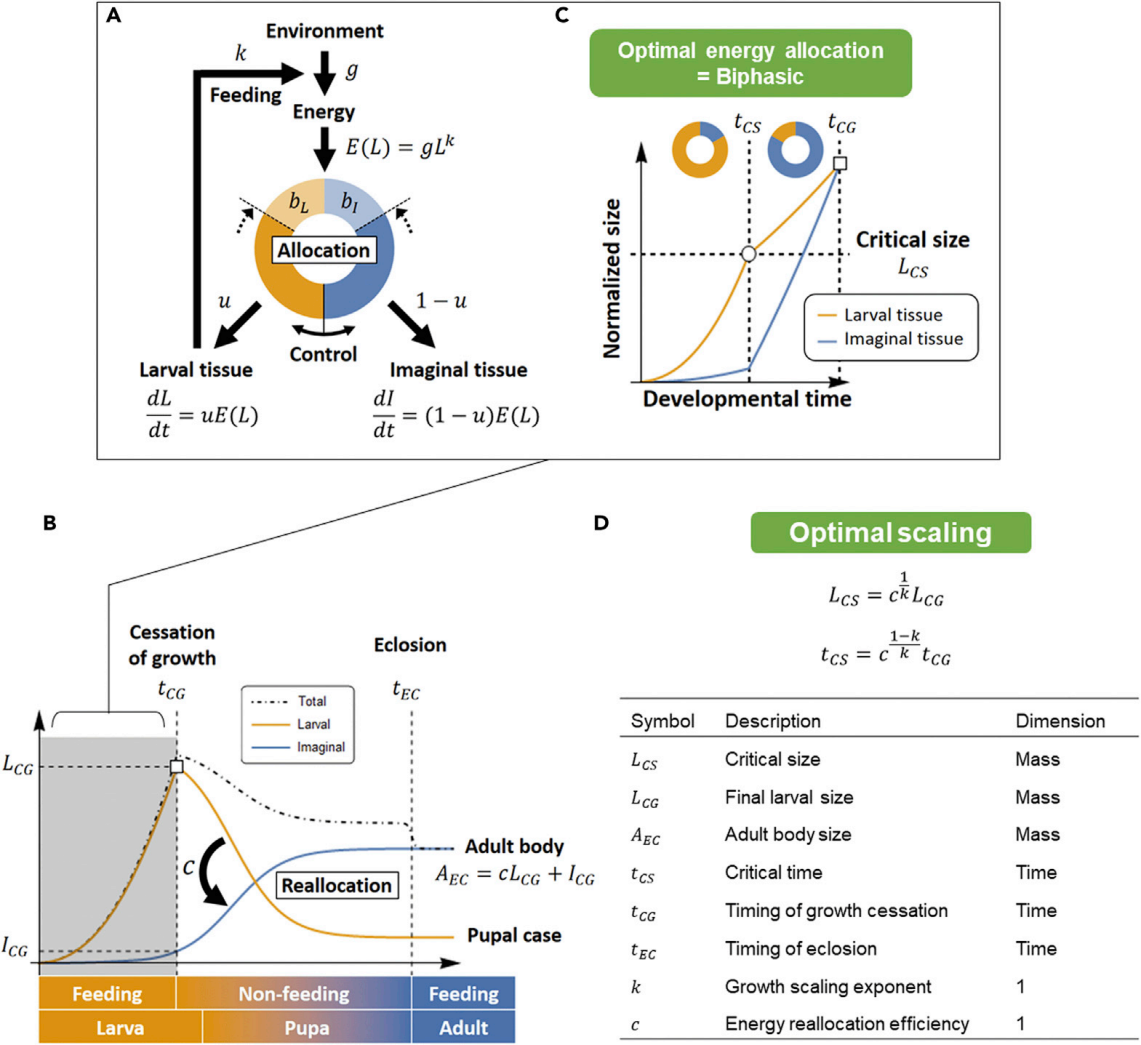
Optimal Life History Model for Holometabolous Insects (A) During the larval stage (*t* ∈ [0,*t*_*CG*_]), the larva acquires energy (*E*) from the environment in a larval body size-dependent manner (*E*(*L*) = *gL*^*k*^) and allocates it to the growth of the larval (*L*, orange) and imaginal tissue (*I*, blue) according to the ratio *u*(*t*):1 − *u*(*t*) and under the constraint *u* ∈ [*b*_*L*_, 1−*b*_*I*_] (see Transparent Methods for details). (B) During the pupal stage (*t* ∈ [*t*_*CG*_, *t*_*EC*_]), there is no feeding and therefore no energy influx from the environment to the pupa. But the imaginal tissues continue growing using energy stored in larval tissues, eventually becoming the body structures of the mature adult. Representing the efficiency with *c*, this energy reallocation process is written as *A*_*EC*_ = *cL*_*CG*_ + *I*_*CG*_. (C) When maximizing fitness (e.g., adult body size) using the bang-bang principle for linear control systems, the energy allocation during the larval stage becomes biphasic: earlier larval growth and later imaginal growth (see Transparent Methods for details). We regard the phase-transition point as the critical size (or critical time; circle). (D) Scaling relationship derived from the optimal life history model. Meanings of variables and parameters are shown in the lower table.

We first introduce the life history model for holometabolous insects and explain how evolution of three size determinants (critical size, TGP, and growth rate) are governed by the optimality principle (see Transparent Methods and Hironaka and Morishita, 2017 for more detailed explanation). The model consists of two state variables: the size of larval tissues, *L*, and the size of imaginal tissues (precursors of adult organs), *I*. During the larval stage (0 < *t* < *t*_*CG*_), the organism acquires food from the environment and metabolizes it into energy and materials. Assuming that this process depends only on larval body size, we can write the energy dedicated to growth as *E* = *gL*^*k*^ (measured in units of body mass per time), where *g* is the assimilation rate coefficient and *k* is the growth scaling exponent (Bonduriansky and Day, 2003, Day and Taylor, 1997, Sears and Kerkhoff, 2012, West et al., 2001). The growth energy *E* is allocated to larval and imaginal tissues according to a controlled ratio, *u*, which is later optimized (Figure 2A). During the pupal stage (*t*_*CG*_ < *t* < *t*_*EC*_), there is no energy influx from the environment to the organism, but imaginal tissues continue to grow using the energy and materials stored in larval tissues, eventually creating the adult body, *A*_*EC*_ (Figure 2B). This energy reallocation process is written as *A*_*EC*_ = *cL*_*CG*_ + *I*_*CG*_ where *c* is the efficiency of energy reallocation.

Under these constraints, when optimizing the energy allocation schedule during the larval stage to maximize adult body size and minimize developmental time, the larval growth pattern inevitably becomes biphasic, due to the bang-bang principle for linear control systems (Hironaka and Morishita, 2017). Specifically, at some time during the larval stage (*t*_*CS*_ ∈ [0,*t*_*CG*_]), the growth of larval tissues will slow and the growth of imaginal tissues will speed up (Figure 2C). Because a similar change in growth pattern is observed at the time of critical size attainment *in vivo* (Bryant and Levinson, 1985, Mirth et al., 2009, Truman et al., 2006), we regard the timing of the switch in energy allocation to be the same as the timing of critical size attainment (“critical time”). Therefore, in our model, the late larval growth period after the critical time (*t*_*CS*_ < *t* < *t*_*CG*_) corresponds to the terminal growth period (TGP). Although the explicit forms of the critical size $L_{CS}:=L\left(t_{CS}\right)$ and critical time *t*_*CS*_ cannot be derived unless the function form of fitness is given, their relative values to the final larval size and to the larval development time can be given, independently of fitness function:

(Equation 1A) $$L_{CS}=c^{\frac{1}{k}}L_{CG}$$

(Equation 1B) $$t_{CS}=c^{\frac{1-k}{k}}t_{CG}$$

Thus, only two dimensionless parameters, growth scaling exponent *k* and energy reallocation efficiency *c*, determine the relative values of critical size and critical time. (Equation 1A), (Equation 1B) insists that the critical size and critical timescale proportionally with the final larval size and larval development time, respectively, as long as the values of *k* and *c* are conserved among focal species. Therefore, we refer to these equations as the “optimal scaling.” The optimal scaling relationship represents a guiding principle for three size determinants: critical size, TGP, and growth rate. A simpler expression of this relationship is derived under the assumption that growth is exponential during TGP, which is often used in other studies (Nijhout et al., 2006, Shingleton et al., 2008, Vollmer et al., 2016):

(Equation 2) $$\left[\text{TGP}\right]\propto {\left[\text{Growth rate}\right]}^{-1}$$

where [TGP] is the duration of TGP and [Growth rate] is the exponential growth rate during the TGP. The detailed derivation of Equation 2 is described in Transparent Methods. This inversely proportional relationship between TGP and growth rate provides insight into the physiological mechanisms by which organisms optimize the energy allocation schedule during evolution, as discussed in detail later.

### The Optimal Scaling of Critical Size Is Experimentally Validated in the Genus *Drosophila*

**Figure 3:**
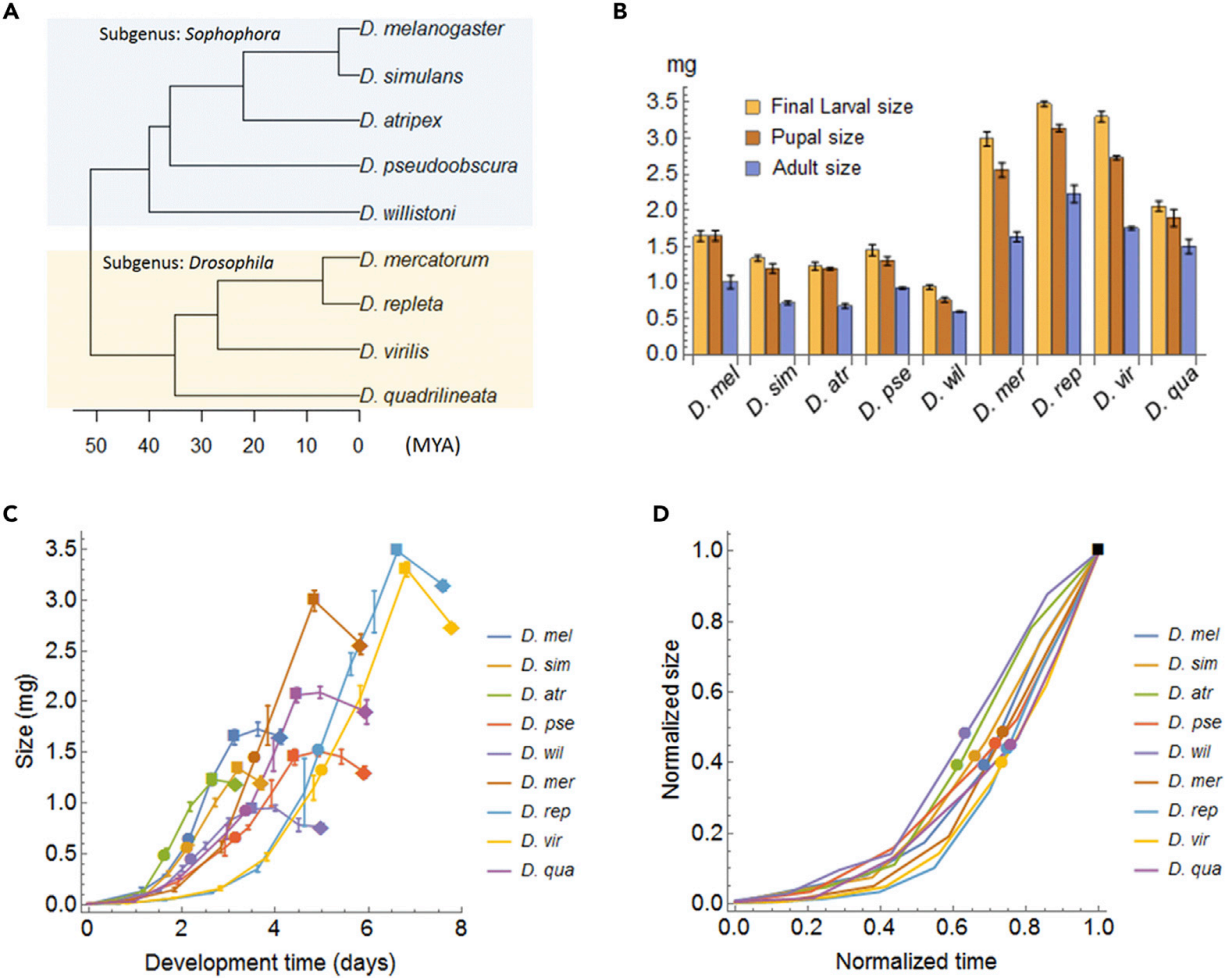
Growth Profiles of the Nine *Drosophila* Species (A) Phylogenetic tree of *Drosophila* species in this study. Branch length indicates evolutionary divergence time, obtained by integrating published data (Grath et al., 2009, Katoh et al., 2007, Song et al., 2011). (B) Larval, pupal, and adult size of the nine species (sampled at growth cessation, pupariation, and eclosion, respectively). Error bars represent standard errors of the mean (n = 3–10 batches). (C) Larval growth curves of the nine *Drosophila* species. After the time of growth cessation (squares), larvae enter the non-feeding/wandering period, leading to pupariation (diamonds). Filled circles indicate the timing of critical size attainment. Error bars represent standard errors of the mean (n = 3–10 batches). (D) All larval growth curves and positions of critical size attainment (filled circles) were mostly overlapping when normalized by the maximal points (square).

A key question concerns whether critical size, TGP, and growth rate are freely determined or governed by the optimal scaling. To this end, we measured interspecific variation in critical size and other growth parameters in nine *Drosophila* species and checked the following three conditions: (1) the proportional relationship between critical size (*L*_*CS*_) and final larval size (*L*_*CG*_), (2) the constancy of *k* and *c* between species, and (3) the consistency between the observed proportionality constant (*L*_*CS*_/*L*_*CG*_) and the predicted proportionality constant (*c*^1/*k*^). Flies used in experiments were from five species of the subgenus Sophophora (*D. melanogaster*, *D. simulans*, *D. atripex*, *D. willistoni*, and *D. pseudoobscura*) and four species of the subgenus Drosophila (*D. mercatorum*, *D. repleta*, *D. virilis*, and *D. quadrilineata*) (Figure 3A).

First, to check the proportional relationship, we measured the final larval size and the critical size for each species. When nine species were raised in a 25°C incubator with a common standard diet, they grew to be normal adults, although development speeds differed between species (Figures 3B and S1). At the timing of growth cessation (*t*_*CG*_), we observed interspecific variation in the final larval size *L*_*CG*_ of up to 3-fold (Figures 3B and 3C). Next, critical size *L*_*CS*_ was measured by starvation experiments (see Methods and Transparent Methods for details). In *Drosophila*, the “minimal viable weight,” a size below which animals cannot survive starvation to pupariate, is often used as a proxy for critical size (method A—Mirth et al., 2005, De Moed et al., 1999, Ohhara et al., 2017). Alternatively, the “breakpoint” in a plot of larval mass at starvation versus time to pupariation, namely, a size below which larvae pupariate with a significant delay, is defined as critical size in several studies (method B—Callier et al., 2013, Ghosh et al., 2013, Stieper et al., 2008, Testa et al., 2013). To examine both methods, we obtained the pupariation rate and pupariation time as functions of the larval size at starvation and calculated the critical size for each species in two different ways (Figures S2 and S3). Both methods provided quantitatively similar results (*r* = 0.99, Pearson's correlation coefficient; Figure S3C); therefore, here we show results of the former method, unless otherwise mentioned.

**Figure 4:**
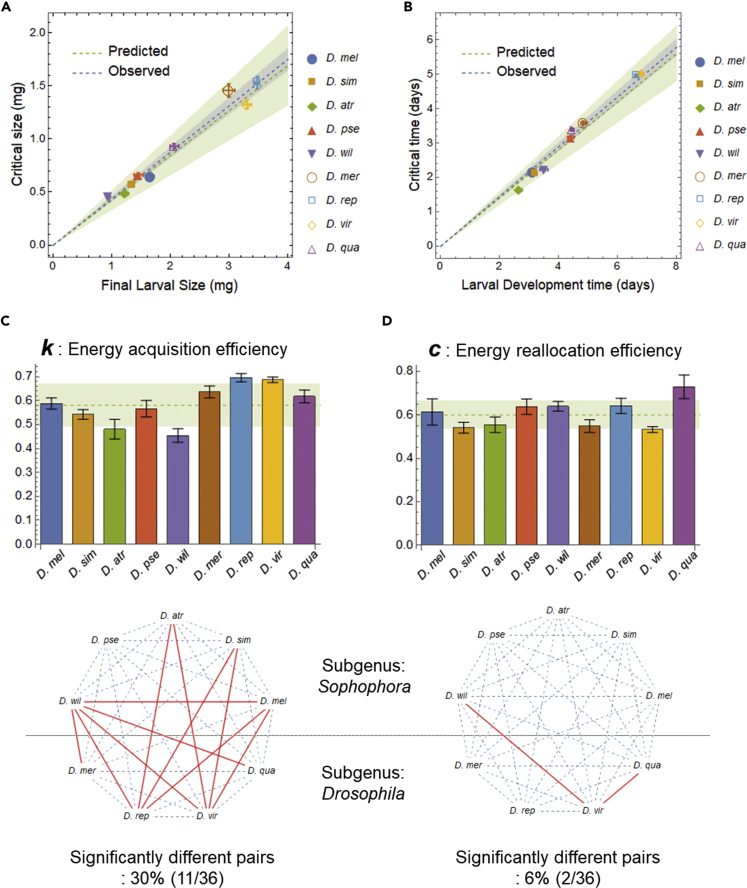
Optimal Scaling of Critical Size and Critical Time (A and B) Scaling relationships between critical size and final larval size (A) and between the larval development time and the critical time (B). Blue lines show the regression lines fitted by the linear model without an offset (blue shaded area: 95% CI), whereas green lines show the theoretical lines predicted by the life history model using the average of *c* and *k* from nine *Drosophila* species (green shaded area: standard deviation between the nine species). Here we used method A to calculate critical size. (C and D) Interspecific comparison of parameters for energy acquisition efficiency *k* (C) and for energy reallocation efficiency *c* (D). Error bars are the standard errors calculated by the law of propagation of uncertainty. In the background, mean (dashed lines) and ±SD (shaded area) of nine species are shown. In lower panels, significantly different pairs are connected by red lines (pairwise z-test comparisons with Bonferroni correction at a significance of *α* = 0.01). Most of the significantly different pairs cross the border of subgenus indicated by the black dashed line.

We found that critical size had an interspecific variation of up to 3-fold, similar to the final larval size (Figures 3C and 3D), and confirmed a proportional relationship between critical size and final larval size ($L_{CS}=0.44*L_{CG},R^{2}=0.99$; blue dashed line in Figure 4A; see Figure S4A for method B), as well as between the critical time and the larval development time ($t_{CS}=0.72*t_{CG},R^{2}=0.99$; blue dashed line in Figure 4B; see Figure S4B for method B). Interestingly, the critical size of the largest species (*D. repleta*) was larger than the final larval size of the smallest species (*D. willistoni*), although larval morphologies of these species are almost indistinguishable. This observation suggests that the critical size is determined not by mechanical limit but by evolutionary consequences.

Second, to check the constancy of *k* and *c* between species, we estimated these parameters for each species by measuring the ontogenetic growth curve. We determined *k* by fitting the larval growth curve with a power law for each species (see Transparent Methods; Figure S1D). All larval growth curves were mostly overlapping when normalized by their maximal points, which indicates small *k* variation (Figure 3D). Indeed, interspecific differences in the value of *k* were non-significant for 70% of all possible pairs (25/36, pairwise z-test comparisons; Figure 4C). Next, we obtained *c* from the ratio of the final larval size to the adult size at eclosion (see Transparent Methods; Figure 3B). Despite substantial variation in body size, interspecific differences for *c* values were non-significant for 94% of all possible pairs (34/36, pairwise z-test; Figure 4D). For both *k* and *c*, which had ranges of possible values between 0 and 1, the intra-genus variations were less than 10%: *k* = 0.58 ± 0.09, *c* = 0.6 ± 0.07 (mean ± SD of nine species; Figures 4C and 4D). Thus, we concluded that both *k* and *c* can be regarded as almost constant between species. These results led us to expect that the optimal scaling of critical size is valid in these species.

Third, to check the consistency between the observed proportionality constant (*L*_*CS*_/*L*_*CG*_) and the predicted proportionality constant (*c*^1/*k*^), we calculated the latter value by using the average values of *k* and *c* of nine *Drosophila* species and compared it with the former value, which is already calculated previously. For the scaling relationship of critical size indicated by Equation 1A, the predicted constant was: *c*^1/*k*^ = 0.42 ± 0.09 (mean ± SD of nine species; the green dashed line and shaded region in Figures 4A and S4A), which was quantitatively consistent with the observed constant: *L*_*CS*_/*L*_*CG*_ = 0.44 ± 0.03 (mean ± 95% confidence interval [CI]; Figure 4A). For the scaling relationship of critical time indicated by Equation 1B, the predicted constant was: $c^{\left(1-k\right)/k}=0.7\pm 0.1$ (mean ± SD of nine species; the green dashed line and shaded region in Figures 4B and S4B), which was also close to the observed constant: *t*_*CS*_/*t*_*CG*_ = 0.72 ± 0.03 (mean ± 95% CI; Figure 4B). Finally, for all pairs of the observed constant and the predicted constant (for critical size or critical time; for method A or method B), we performed equivalence tests using two one-sided z-tests (Hatch, 1996). In each of the four comparisons, the null hypothesis |Δx|>δ was rejected at a significance level of 0.01, where $\text{Δ}x:=x_{\text{observed}}-x_{\text{predicted}}$ is the difference between observation and prediction and *δ* is the standard deviation of *x*_predicted_ (the standard error of *x*_observed_ was used as that of Δ*x*). These results indicate that the observed values are within the theoretically expected range: *x*_predicted_ − *δ* ≤ *x*_observed_ ≤ *x*_predicted_ + *δ*.

We should note that, even if we assume that the proportionality constants differ between species, observed values for relative critical size and for relative critical time show good agreement with the predicted values (Figures S4C and S4D).

Taken together, we confirmed (1) the proportional relationship, (2) the constancy of *c* and *k*, and (3) the consistency between the observed proportionality constant and the predicted proportionality constant. These results indicate that the life history model is accurate to experimental observations, at least in the genus *Drosophila*, and suggest that size determinants, including critical size, TGP, and growth rate, are constrained by the optimal scaling.

### The Scaling of Critical Size Is Achieved by an Evolutionary Change that Inversely Correlates Growth Duration and Growth Rate

**Figure 5:**
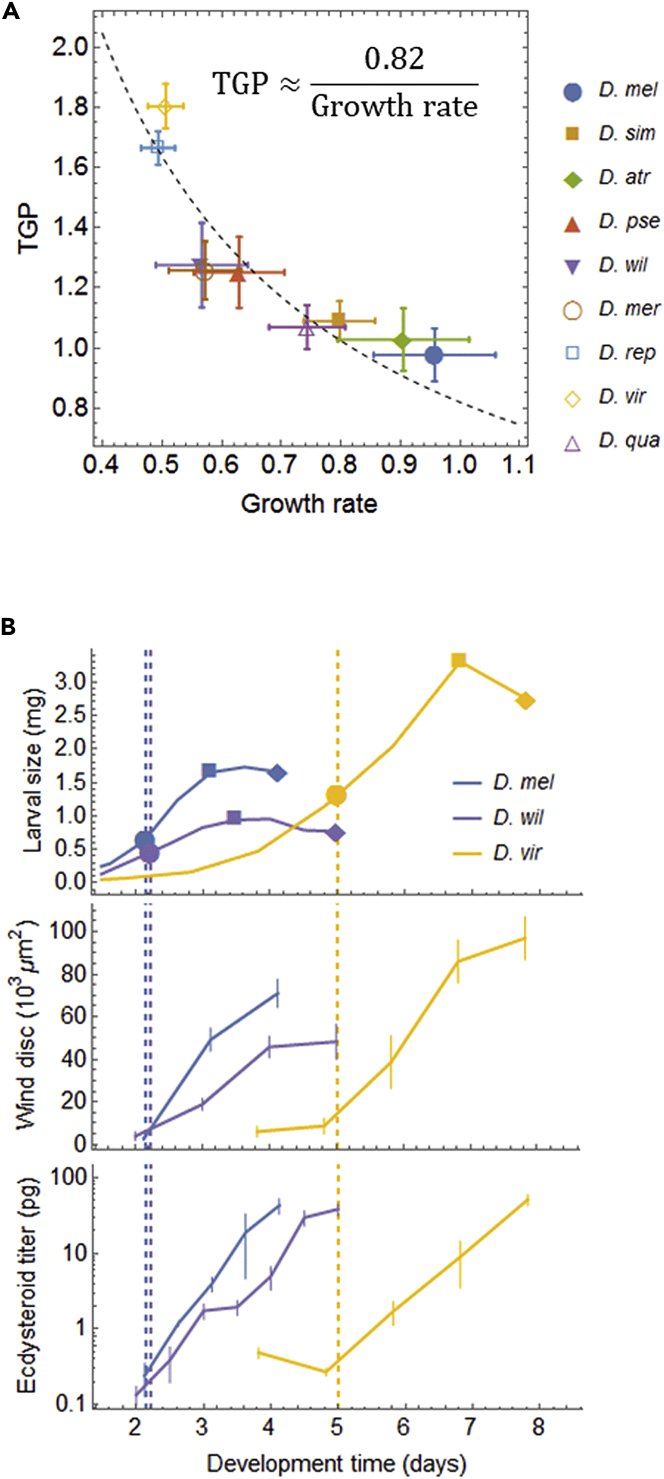
Mechanisms Underlying the Optimal Scaling of Critical Size (A) In the nine species, there is a negative correlation between the growth rate and the duration of TGP, which ensures the scaling between critical size and final size. The dashed curve indicates the nonlinear regression fit (*y* = 0.82/*x*). Error bars are standard error calculated by the law of propagation of uncertainty. (B) For the three species with marked differences in the TGP duration (*D. mel*, *D. wil*, *D. rep*), growth curves of larval body (upper panel) and wing imaginal disc (middle panel) and temporal profiles of ecdysteroid titer (lower panel) are shown. Filled circles, squares, and diamonds indicate the timing of critical size attainment, growth cessation, and pupariation, respectively. Error bars represent standard deviations (n = 3–6 batches).

As explained earlier, the inversely proportional relationship between TGP and growth rate is theoretically derived from the optimal scaling between critical size and final size. Indeed, we observed a strong negative correlation between the TGP and growth rate (*r* = −0.85; Figure 5A), which can be accurately approximated by the inverse proportionality: $\left[\text{TGP}\right]=0.82*{\left[\text{Growth rate}\right]}^{-1}$ (Figure 5A). From these observations, we can infer that, during evolution, organisms optimized the energy allocation schedule by adaptively changing TGP for the species-specific growth rate.

Then, we asked what molecular mechanisms determine the interspecific difference in the TGP. Because the duration of TGP (or the timing of growth cessation) is regulated by ecdysteroid (Yamanaka et al., 2013), we investigated the dynamics of ecdysteroid titer in three flies that had marked differences in TGP duration (*D. melanogaster, D. willistoni, D. virilis*). We also measured growth curves for the wing imaginal disc as another index of developmental progression. All species had exponential increases in both ecdysteroid titer and growth of the imaginal disc after reaching critical size (Figure 5B), consistent with our model's predictions (Figure 1C). There was clear interspecific variation in the rate of increase of ecdysteroid titer, which positively correlated with the TGP duration; the species with a shorter TGP had faster rates of ecdysteroid increase. These results imply that the change in hormone dynamics caused the change in developmental rate during divergence of these species (i.e., heterochrony).

Finally, we asked which molecular mechanisms underlie the correlation between the ecdysteroid synthesis rate and growth rate. It is possible that this correlation is due to a built-in mechanism in *Drosophila*, because a negative correlation between TGP and the growth rate is also observed in sexual dimorphism (Testa et al., 2013) and many types of phenotypic plasticity in response to temperature (Ghosh et al., 2013), oxygen (Callier et al., 2013), and nutrition (Layalle et al., 2008). In particular, Layalle et al. (2008) reported that larvae reared with low nutrition had a decreased growth rate and an increased TGP, both of which are regulated by insulin/insulin-like growth factor signaling (IIS) and/or TOR signaling (Layalle et al., 2008): a systemic decrease in IIS and/or TOR signaling downregulates the organismal growth rate through peripheral tissues, including the imaginal disc and fat body. In contrast, decreased IIS and/or TOR signaling in the prothoracic gland that governs ecdysteroid production prolongs the duration of TGP. Thus, because IIS and/or TOR signaling can inversely regulate growth rate and growth duration, these may serve as an underlying mechanism for scaling critical size during evolution. Although optimal scaling in a broad sense (indicated by (Equation 1A), (Equation 1B)) cannot predict which of three size determinants contribute to final size evolution, if such a built-in scaling mechanism is conserved across species, critical size would be a main driver of final size evolution. Future work should compare the intensity of insulin signaling between different species and clarify the potential roles of IIS and/or TOR signaling in critical size scaling and final size determination.

### Applicable Scope of the Optimal Scaling and Energy Allocation Switch

The high coefficient of determination (*R*^2^ = 0.99 in Figures 4A and 4B) in the optimal scaling relationship indicates that body size difference at the interspecific level can be explained solely by the critical size. The inverse relationship between TGP and growth rate clearly explains why these two factors do not contribute to body size difference, whereas their values differ between species: the opposite directions of their variation cancel out each other's effect on final size. Although this is seemingly contrary to the conventional view that critical size, TGP, or growth rate can contribute to the determining final size (D’Amico et al., 2001, De Moed et al., 1999, Nijhout et al., 2006, Partridge et al., 1999), it is a matter of scale: if the genetic distance is too small, it would be difficult to recognize the scaling relationship because the size variation would be too small. Conversely, if the genetic distance is too large, the scaling relationship would not hold because physiological parameters such as *c* and *k* can differ between species. What we observe from an interspecies or interstrain comparison can change depending on the focal scale of evolutionary time or genetic distance.

### Limitation of This Study

We should note that not all holometabolous insects commit to becoming pupae in a manner dependent on critical size, like *Drosophila*. In some beetles and solitary bees, critical size does not seem to exist and pupation is triggered by food deprivation or starvation (Helm et al., 2017, Nagamine et al., 2016, Nijhout, 2008). Even in *Manduca sexta*, which does have a critical size, the pupation mechanism has several physiological features that are distinct from *Drosophila* (e.g., inhibitory effect of juvenile hormone on pupation, log-linear relationship between molting size and critical size; please see Transparent Methods and Figure S5 for details). These observations suggest that the pupal commitment mechanisms are very diverse between species, but the concept of an energy allocation switch can be applied more broadly across species and apart from critical size. Even in insects without a critical size, the pupal commitment determining final body size may still be closely linked to an energy allocation switch (Figure 2).

### Conclusion

Our data show that the critical sizes of nine *Drosophila* species follow a scaling relationship that is derived from an optimal energy allocation model. Furthermore, we find that an inverse correlation between growth rate and growth duration, which is caused by temporal scaling of hormone dynamics, cancels out their effects on the final size and makes critical size contribute more directly to the final size. These findings emphasize the central role of critical size in size determination and suggest a common principle for the evolution of organism size. Similar principles may be found in organisms other than holometabolous insects by developing an energy allocation model that accounts for organism-specific life history.

## Methods

All methods can be found in the accompanying Transparent Methods supplemental file.

## Supplemental Information

Document S1. Transparent Methods, Figures S1–S5, and Table S1
