# Supplementary material for: Optimal Scaling of Critical Size for Metamorphosis in the Genus Drosophila
Source: iScience. 2019 Sep 27;20:348–58. doi: 10.1016/j.isci.2019.09.033 (PMC6817650; doi:10.1016/j.isci.2019.09.033)
Supplement: Document S1. Transparent Methods, Figures S1–S5, and Table S1 [file mmc1.pdf]

ISCI, Volume *20*

Supplemental Information

Optimal Scaling of Critical Size for Metamorphosis in the Genus *Drosophila*

Ken-ichi Hironaka, Koichi Fujimoto, and Takashi Nishimura

Supplementary figures

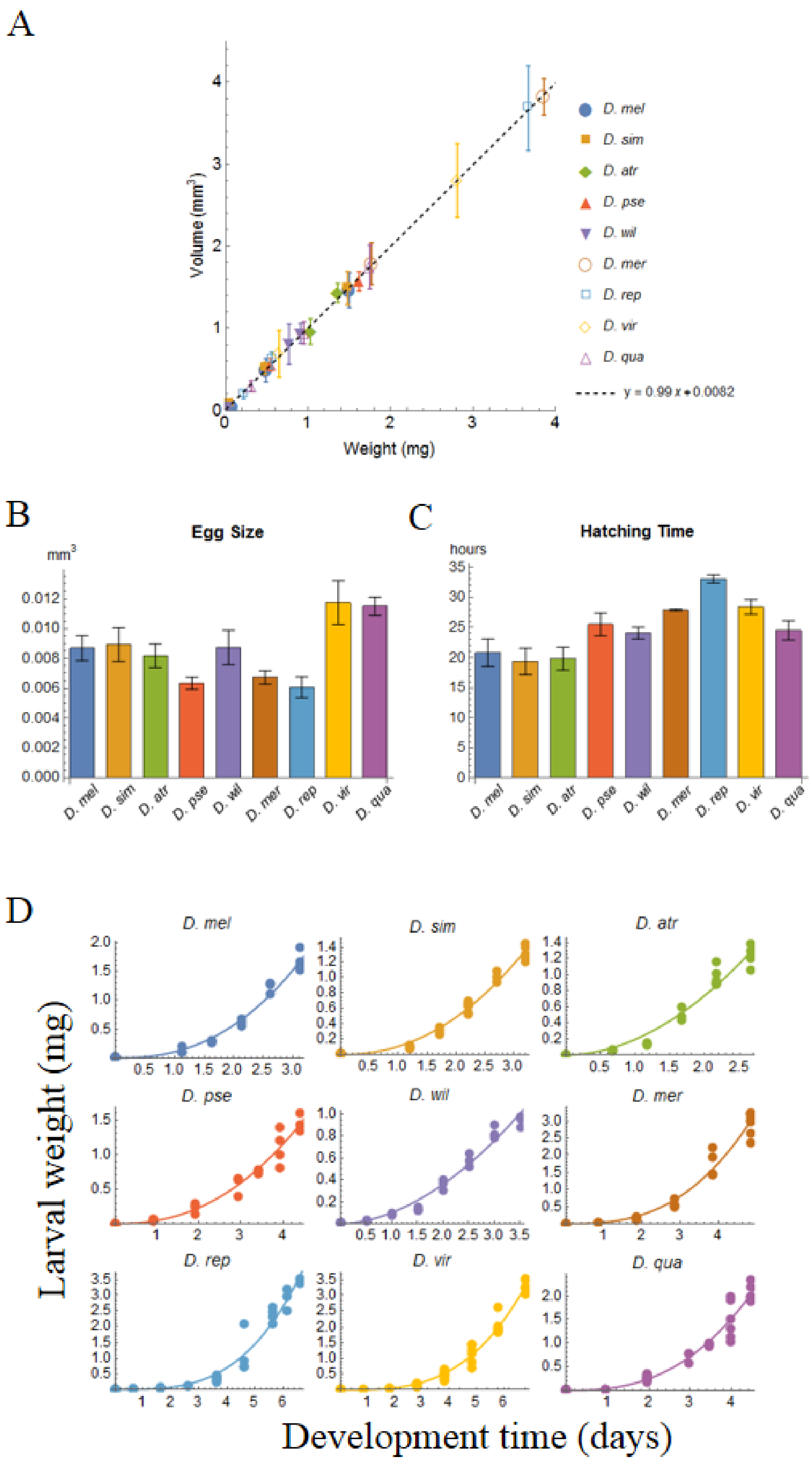

Figure S1. Egg size and hatching time of the nine *Drosophila* species, related to Figure 3.

(A) Larval weight (mg) vs. larval volume (mm^3). Data points around the diagonal $(y=x)$ indicates that the density of larvae is almost the same as the density of water (1 $\mathrm{g/cm^3} = 1\ \mathrm{mg/mm^3}$). Error bars represent the standard deviations (n = 10). **(B)** Egg volume (V) estimated by the formula of a prolate spheroid: $V = 4/3\pi(L/2)(l/2)^2$ (L is the length and l is the width of egg, measured under the microscope). Error bars represent standard deviations (n = 10–21). **(C)** Hatching time measured by time-lapse imaging of eggs reared at 25°C. Error bars represent standard deviations (n = 12–48). **(D)** Fitting of growth curve (from hatching until growth cessation) with a power function $y = ax^b$. The values of parameter k are obtained by $k = 1 - 1/b$.

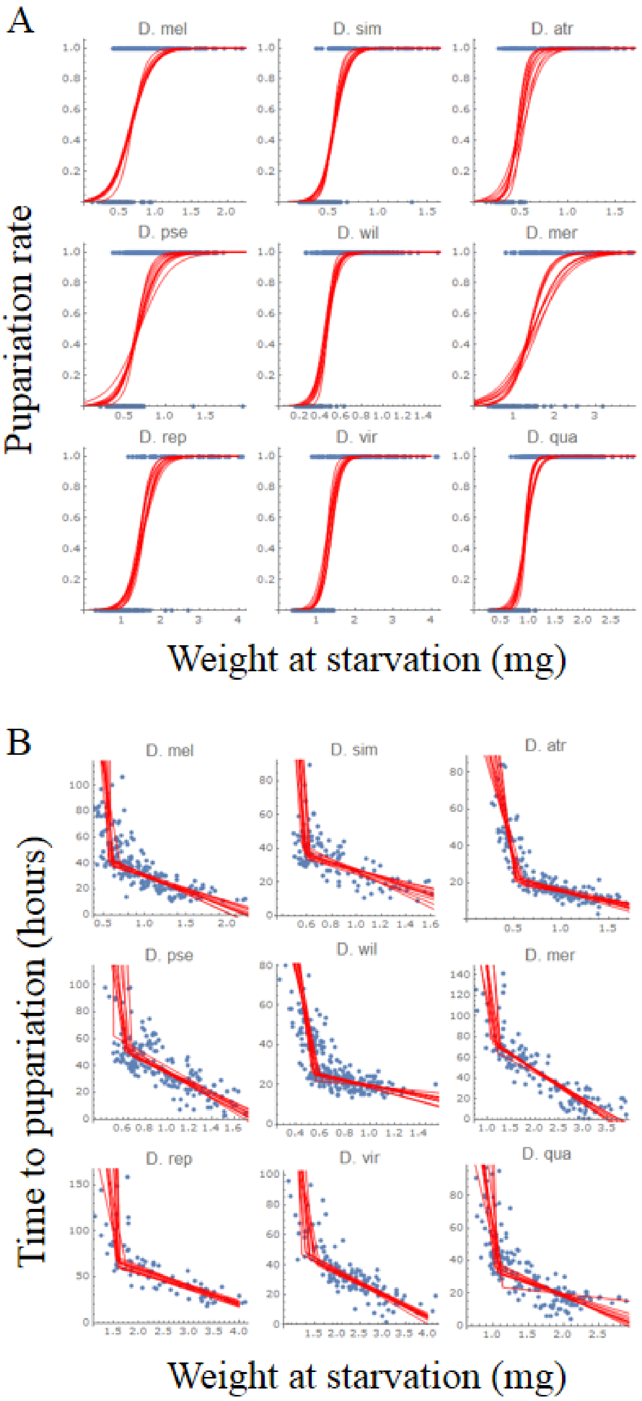

Figure S2. Estimation of critical size, related to Figures 3 and 4.

(A) Method A: logistic regression of the pupariation rate (0 = not pupariated, 1 = pupariated) vs. the weight at starvation. In the regression curves, the weight at which 50% of larvae pupated corresponded to the points at which larvae reached critical size. Regression curves (red) for 10 of the 1000 bootstrapped samples are superimposed on raw data (blue dots). **(B)** Method B: a segmented linear regression of the time to pupariation vs. the weight at starvation. The breakpoints in the regression

lines corresponded to the points at which larvae reached critical size. Regression lines (red) for 10 of the 1000 bootstrapped samples are superimposed on raw data (blue dots).

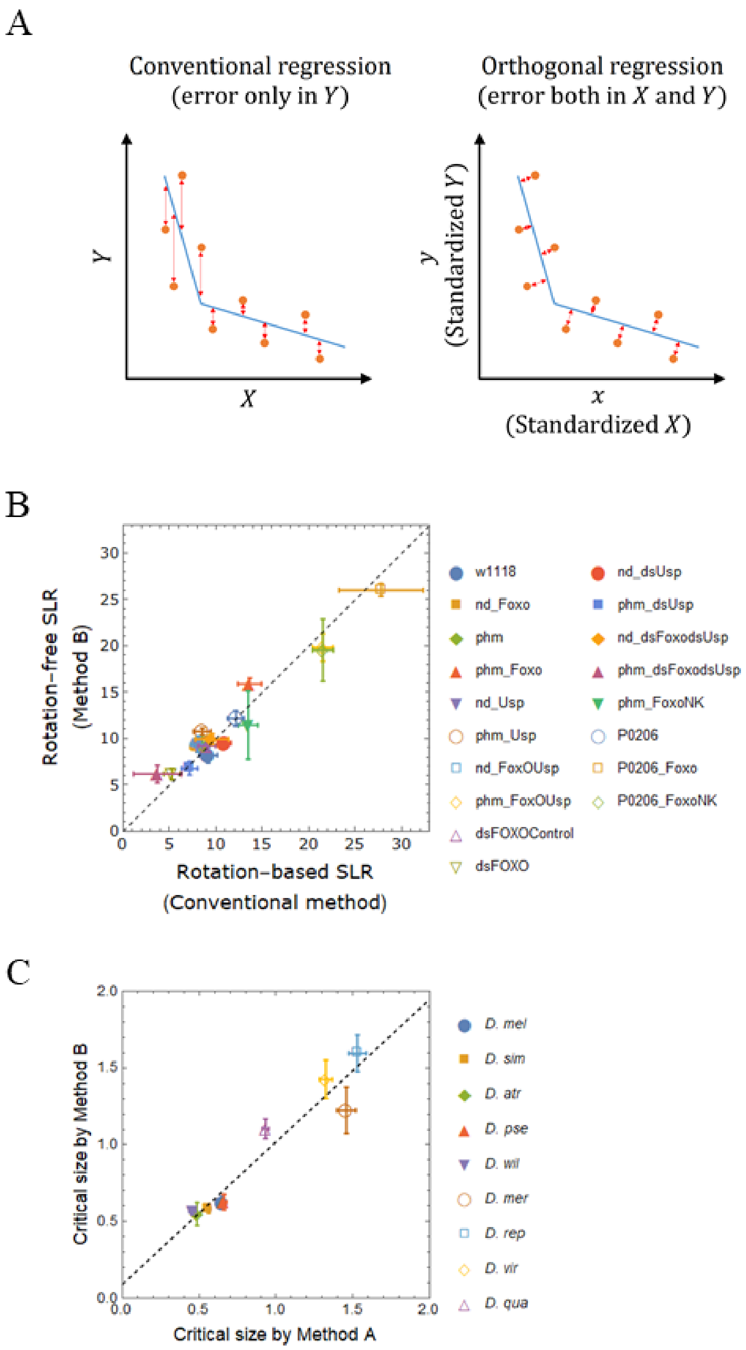

Figure S3. Rotation-free segmented linear regression, related to Figures 3 and 4.

(A) The conventional segmented linear regression considers the errors only in the y-axis (left panel), whereas our method considers the errors in both the x- and y-axes (right panel). **(B)** Breakpoint comparison calculated by two different methods: horizontal axis, rotation-based SLR proposed in previous study (Callier et al., 2013; Ghosh et al., 2013; Testa et al., 2013); vertical axis, rotation-free SLR proposed in this study. Because points appear around the diagonal line for all genotypes, we can see that the method returns almost the same result as the conventional method. Here we used the

published data from the previous study on critical size (Koyama et al., 2014). In this plot, both x and y indicate critical time (hours after L3 ecdysis). **(C)** Comparison of critical weight of nine Drosophila species between methods A and B. The dashed line calculated by linear regression $(0.9x + 0.09)$ represents a significant correlation $(r = 0.96; P < 0.01)$.

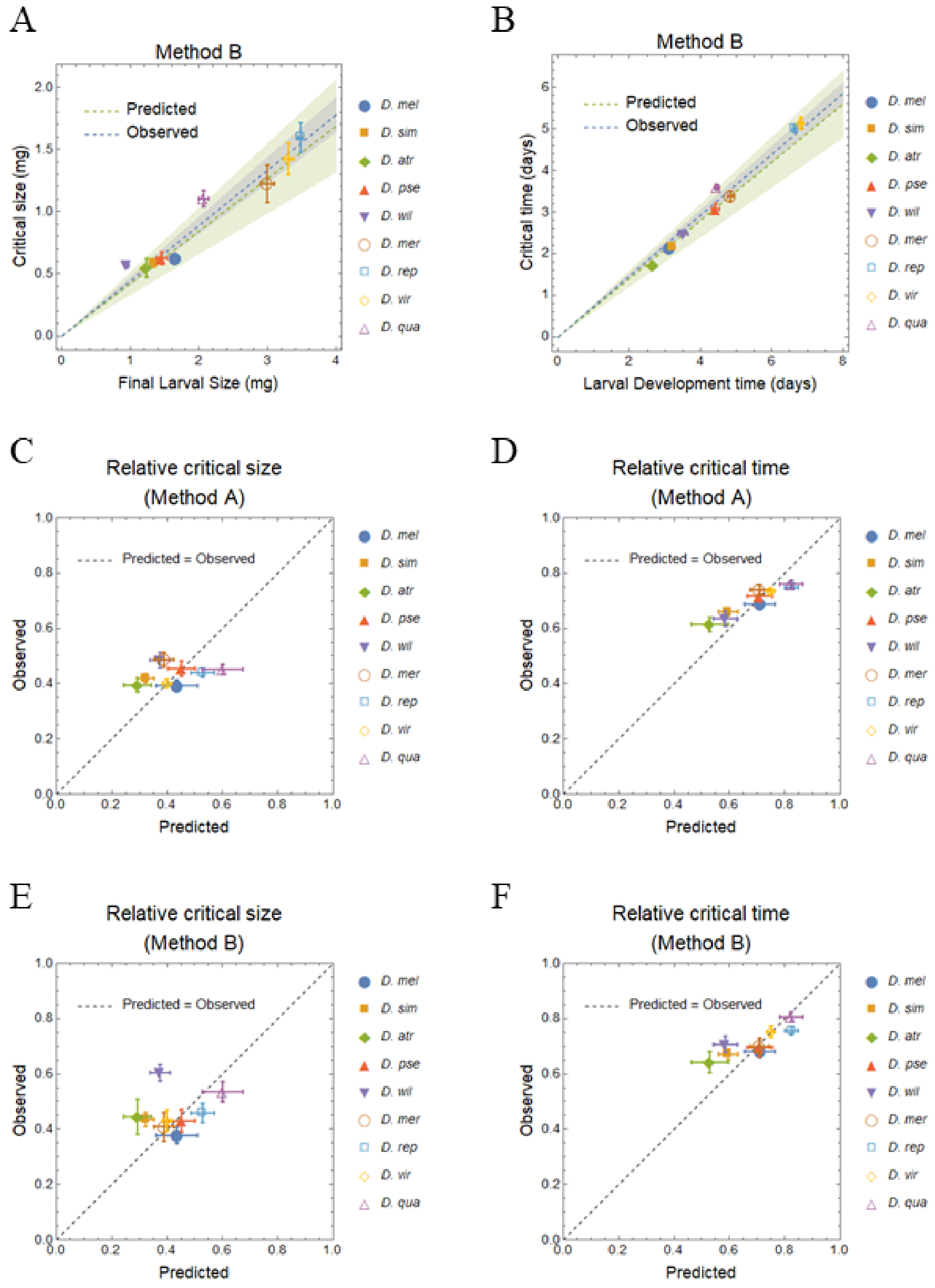

Figure S4. Scaling relationships of critical size and critical time, related to Figures 3 and 4.
(A and B) Scaling relationships between critical size and final larval size (A), and between the larval development time and the critical time (B). Blue lines show the regression lines fitted by the linear

model without an offset [$L_{CS} = 0.45\ L_{CG}$ for (A) and $t_{CS} = 0.73\ t_{CG}$ for (B); shaded area: 95% CI], whereas green lines show the same result as Figure 4A and B. Here we used method B to calculate critical size. **(C and D)** Comparison of relative critical size (C) or relative critical time (D) between prediction based on method A and experimental observation. **(E and F)** Comparison of relative critical size (E) or relative critical time (F) according to prediction based on method B and experimental observation. In both methods, relative critical size showed weak correlation (method A: 0.24, method B: 0.21) and relative critical time showed strong correlation (method A: 0.95, method B: 0.84). This is probably due to the convexity of growth curve ($d \log L / d \log t < 1$) which caused the measurement error in size to be larger than the measurement error in time, and the smallness of interspecies variation in the proportionality constant compared to the measurement error in size. The dashed diagonal line in (C - F) indicates a perfect match between prediction and observation. All error bars are the standard errors calculated by the law of propagation of uncertainty.

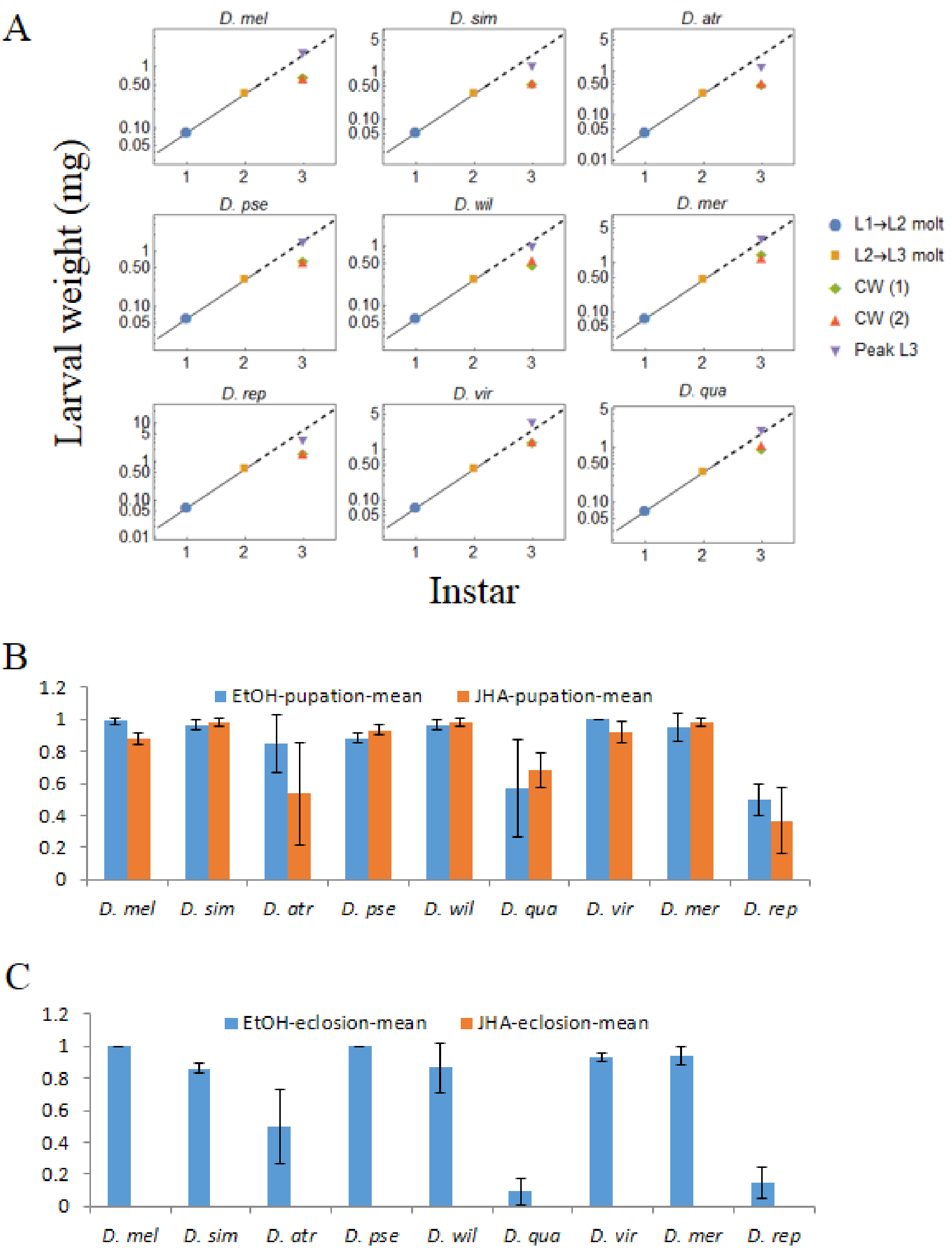

Figure S5. Differences in pupal commitment mechanisms between *Drosophila* and *Manduca*, related to Figure 4.

(A) Relationship between size at molting and critical weight (Dyar's rule). In *Manduca*, critical weight can be predicted by extrapolating the log-linear relation of larval size at molting between each instar (Dyar's rule). However, values predicted in this way were significantly larger than the experimentally observed critical weight of the nine *Drosophila* species in this study. Rather, the peak size of feeding larvae was consistent with this relationship. **(B and C)** Effects of juvenile hormone on pupation rate and eclosion rate. Comparison of (B) pupation rate and (C) eclosion rate between the EtOH-treated

group and JHA-treated group in each species. Each bar indicates mean ± SD of triplicates of 20-30 animals. There was no significant difference in pupation rate between the two groups ($p = 0.21$, two-way ANOVA), whereas the eclosion rate was significantly different between the two groups ($p < 0.01$, two-way ANOVA). We did not observe an eclosed adult in the JHA-applied group for any species.

Supplementary table

Table S1. Data of *Manduca sexta*, related to Figure 4.

	Predicted ($c = 0.24, k = 0.94$)	Observed (Minimum viable weight)	Observed (Critical weight)
Relative size	0.21	0.29	0.63
Relative timing	0.91	0.86	0.92

Transparent Methods

Fly stocks and rearing

In this study, Oregon R (*OreR*) strain was used as wild-type for *D. melanogaster*, and other species (*D. simulans, D. atripex, D. willistoni, D. pseudoobscura, D. quadrilineata, D. virilis, D. mercatorum,* and *D. repleta*) were derived from strains maintained in EHIME-fly, a laboratory for *Drosophila* resources at Ehime University. All flies were reared at 25°C on standard food containing 8 g of agar, 100 g of glucose, 45 g of dry yeast, 40 g of corn flour, 4 ml of propionic acid, 0.45 g of butylparaben (in ethanol) per liter.

Measurement of growth curves

After a 2-4 h egg-laying period, eggs were transferred onto the food to maintain a density of 20-40 individuals per vial. Then, after a defined feeding period, flies of mixed sexes were weighed in more than three batches of about 10 individuals (or 50 individuals for 1st instar larvae) for each time-point. Larvae and pupae were washed with phosphate-buffered saline and carefully dried with a Kimwipe before weighing. Adults were weighed after being anaesthetized with carbon dioxide. We calculated "time after hatching" (x-axis of growth curve) by subtracting "average hatching times" from "time after egg laying" (hatching time was measured by time-lapse imaging of eggs with a digital camera). Timing of "cessation of growth" was defined as the point when weight no longer increased by 10% or more since the previous time-point. For the growth curve of the imaginal disc in **Figure 5B**, the wing disc was giemsa-stained, photographed with a Zeiss Primo Star microscope equipped with a AxioCam ERc 5s digital camera (Zeiss), and its area was measured by Image J.

Starvation experiments

For each species, the mixed-sex third instar larvae of various sizes were removed from the food source and photographed under a stereomicroscope (Zeiss) with a digital camera (Canon) to quantify their size. We determined the volume of the larvae using the prolate spheroid formula $4/3\pi(L/2)(l/2)^2$ (where L is the length and l is the width). Note that the volume (mm^3) and mass (mg) can be converted to each other by using water density ($1\ \mathrm{mg/mm}^3$), as shown in Figure S1A. Then, each measured sample was transferred to one well of a 48-well plate filled with 0.8% agar medium to prevent dehydration. The agar plate on which larvae were transferred was placed in a 25°C incubator and photographed every hour with a Canon EOS Kiss X7 digital camera (Canon). After 10 days, we checked whether each larva became a pupa or not (data for method 1). If it became a pupa, we recorded the time taken to pupariate from the time-lapse images (data for method 2).

Calculating critical size

From the starvation experiment, we calculated critical size using two alternative statistical methods (method A, based on pupariation rate; or method B, based on pupariation time). Method A addresses whether the larvae became pupae or not, as a binary function of larval size (0 = not pupariated, 1 = pupariated) and uses logistic regression to detect "a size that entails 50% probability of becoming a pupa," which is regarded as a critical size (De Moed et al., 1999; Mirth et al., 2005; Ohhara et al., 2017). Method B addresses the time to pupariation as a function of larval size and uses segmented linear regression (SLR) to detect a breakpoint, wherein the x-value is regarded as a critical size (Callier et al., 2013; Ghosh et al., 2013; Stieper et al., 2008; Testa et al., 2013). Because it is technically difficult for a conventional SLR method to detect breakpoints close to the end points of data, we developed a SLR method that can robustly detect breakpoints (SI Text S3). For both methods A and B, we calculated the average and standard deviation of critical size from 1000 bootstrapped samples of each species.

Calculating molting size

For each species, mixed-sex larvae of various instar stages were taken from the food source and their volume was measured in the same manner described above. We determined larval stages by observing spiracle and mouth hook morphology (Ashburner et al., 2005). We treated whether the larva were the (n)th instar or the (n+1)th instar as a binary function of larval size [(n)th = 0, (n+1)th = 1], and used logistic regression to detect "size at the time when there was a 50% mix of (n)th and (n+1n)th larvae " which is regarded as the molting size of (n)th instar (n = 1, 2).

Application of JHA

For each species, the feeding third instar larvae of mixed sexes were taken from the food source, divided into two groups of the same size (20-30 animals), and each group was transferred to a new food source. On a new food source, 30 μl of the JH analog methoprene (SIGMA, diluted to 10 ppm with ethanol) was topically applied to one group, and the same amount of ethanol was applied as a control to the other group. For each group, we measured the rate of pupation and eclosion. Experiments were repeated three times and statistical tests were performed by two-way repeated ANOVA.

Quantification of ecdysteroids by LC-MS/MS

Quantification of ecdysteroids was performed as previously described (Lavrynenko et al., 2015) with some modifications. Frozen samples in 1.5 ml plastic tubes were homogenized in 200 μl cold methanol containing 100 pg muristerone A (MuA, internal standard) with 1x ϕ 3 mm zirconia beads using a freeze crusher (Tokken Inc.). The homogenates were mixed with 100 μl of methanol, 100 μl of H_2O, and 100 μl of $CHCl_3$, and vortexed for 20 min at RT. The samples were centrifuged at 15,000 rpm (20,000 *g*) for 15 min at 4°C. The pellets were washed with 300 μl ethanol, re-dissolved with 300

μl of 0.2 N NaOH, and used to quantify protein using a BCA protein assay kit (Thermo). The supernatant was mixed with 200 μl of H_2O and vortexed for 10 min at RT. The samples were centrifuged at 15,000 rpm for 15 min at 4°C, and the aqueous phase was collected and dried down in a vacuum concentrator. The dried material was re-dissolved in 300 μl of 20% methanol. The samples were loaded on MonoSpin C18 columns (GL Sciences Inc.). The columns were pre-washed with 200 μl methanol and water. Upon sample loading and centrifuged for 1 min at 4,000 g, columns were washed with 300 μl 20% methanol. The samples were eluted with 300 μl of 60% methanol. The eluates were dried down, re-dissolved in 10% methanol, and analyzed by Liquid Chromatography with tandem Mass Spectrometry (LC-MS/MS).

Chromatographic separation was performed on an ACQUITY BEH C18 column (50 mm x 2.1 mm, 1.7-μm particles, Waters) in combination with a VanGuard precolumn (5 mm x 2.1 mm, 1.7-μm particles) using an Acquity UPLC H-Class System (Waters). The mobile phase delivered at a flow rate of 0.25 ml/min at 40°C, consisting of solvent A: 0.1% formic acid in acetonitrile, and solvent B: 0.1% formic acid in H_2O. Linear gradients were as follows: 15% A at 0-0.5 min, 15-40% A at 0.5-2 min, 40% at 2-2.5 min, and 15% at 2.5-5 min. The mass spectrometric analysis was performed using a Xevo TQD triple quadrupole mass spectrometer (Waters) coupled with an electro-spray ionization source in the positive ion mode. The MRM transitions were as follows: 20E, *m/z* 481.2 -> 165.2 and 371.3; MuA, *m/z* 497.3 -> 297.2 and 425.3. We optimized analytical conditions using standards solution of 20E (Sigma) and MuA (AG Scientific). Sample concentrations were calculated from a standard curve obtained from serial dilution of each standard, and then normalized to an internal standard and protein values.

Derivation of optimal scaling relationship

For the feeding larval stage ($t \in [0, t_{CG}]$), the growth dynamics of larval and imaginal tissue sizes (L and I, respectively) obey the following equation (Figure 2A):

$$\frac{dL}{dt} = u(t)E(L) \quad \text{[S1A]}$$

$$\frac{dI}{dt} = \big(1 - u(t)\big)E(L), \quad \text{[S1B]}$$

where $E(L) \coloneqq gL^k$ is the total amount of energy (available for growth) obtained from the environment by larval feeding. The parameters g and k are the assimilation rate coefficient and growth scaling exponent, respectively (Bonduriansky and Day, 2003; Day and Taylor, 1997; Hou et al., 2008). The acquired energy is allocated to the growth of larval and imaginal tissue according to a

ratio of $u: 1 - u$ under a constraint ($b_L \leq u \leq 1 - b_I$, assuming that fixed ratio of energy b_L and b_I are always allocated to the larval and imaginal tissue, respectively).

For the non-feeding larval/pupal stage ($t \in [t_{CG}, t_{EC}]$), the size of larval and imaginal tissues at the cessation of net growth (L_{CG} and I_{CG}, respectively) is summed up to the adult body size A_{EC} (Figure 2B):

$$A_{EC} = cL_{CG} + I_{CG}, \tag{S2}$$

where c $(0 < c < 1)$ is the efficiency of energy reallocation.

The growth schedule, which is governed by the energy allocation time course $u(t)$, is optimized for the entire larval period, based on the idea that the life history appears as a result of adaptive evolution (Roff, 2002). The fitness maximized here is assumed to be an increasing function of the adult size: $\phi = \phi(A_{EC}), d\phi/dA_{EC} > 0$ (for more general fitness, please see (Hironaka and Morishita, 2017)). Using Pontryagin's maximum principle, we can find that the optimal energy allocation u^* is biphasic. This is the so-called “bang-bang” control, which is the common solution for linear control systems (Kirk, 2012). Namely, u^* takes the upper limit value $1 - b_I$ until a certain switching time t_{CS}, and thereafter takes the lower limit value b_L:

$$u^*(t) = \begin{cases} 1 - b_I & 0 < t < t_{CS} \\ b_L & t_{CS} < t < t_{CG} \end{cases} \tag{S3}$$

Furthermore, Pontryagin's maximum principle can also instantly lead to the optimal scaling relationship of critical size (Hironaka and Morishita, 2017). Alternatively, assuming the bang-bang control (Eq. S3), we can derive the optimal scaling by standard differential calculus. Hereafter we describe the latter procedure. First, substituting Eq. S3 into Eqs. S1, the growth curve of each tissue can be written as follows:

$$L(t) = \begin{cases} \left[L_0^{1-k} + g(1-k)(1-b_I)t\right]^{\frac{1}{1-k}} & 0 < t < t_{CS} \\ \left[L_{CS}^{1-k} + g(1-k)b_L t\right]^{\frac{1}{1-k}} & t_{CS} < t < t_{CG} \end{cases} \tag{S4A}$$

$$I(t) = \begin{cases} I_0 + \frac{b_I}{1-b_I}(L(t) - L_0) & 0 < t < t_{CS} \\ I_{CS} + \frac{1-b_L}{b_L}(L(t) - L_{CS}) & t_{CS} < t < t_{CG} \end{cases} \tag{S4B}$$

where L_0 and I_0 are parameters describing initial size of larval and imaginal tissue, respectively. We also introduced the notation $L_{CS} \coloneqq L(t_{CS})$ and $I_{CS} \coloneqq L(t_{CS})$ to denote the size of larval and imaginal tissue at critical time, respectively. Now let us remind that the fitness to be maximized can be written as follows:

$$\phi = \phi(A_{EC}) = \phi(L_{CG}, I_{CG}) \quad \text{[S5]}$$

where $L_{CG} \coloneqq L(t_{CG})$ and $I_{CG} \coloneqq L(t_{CG})$. Note that both L_{CG} and I_{CG} can be regarded as functions of the switching time t_{CS}: $L_{CG} = L_{CG}(t_{CS})$, $I_{CG} = I_{CG}(t_{CS})$. Thus, in order for t_{CS} to optimize ϕ, the following condition must be satisfied:

$$\frac{d\phi}{dt_{CS}} = \frac{\partial\phi}{\partial L_{CG}}\frac{dL_{CG}}{dt_{CS}} + \frac{\partial\phi}{\partial I_{CG}}\frac{dI_{CG}}{dt_{CS}} = 0 \quad \text{[S6]}$$

Solving this equation using Eqs. S4, we obtain the scaling relationship between the critical size and the final larval size:

$$\frac{L_{CS}}{L_{CG}} = \left[1 - b_L\left(1 - \frac{\partial_L\phi}{\partial_I\phi}\right)\right]^{\frac{1}{k}} \quad \text{[S7]}$$

where $\partial_L\phi \coloneqq \partial\phi/\partial L_{CG}$ and $\partial_I\phi \coloneqq \partial\phi/\partial I_{CG}$. Moreover, assuming that larval tissue size immediately after hatching is negligible $(L_0 \approx 0)$, the above equation leads to the equation that describes the scaling relationship between the critical time and the total development time:

$$\frac{t_{CS}}{t_{CG}} \approx \frac{b_L}{b_L + (1 - b_I)\left\{\left[1 - b_L\left(1 - \frac{\partial_L\phi}{\partial_I\phi}\right)\right]^{\frac{k-1}{k}} - 1\right\}} \quad \text{[S8]}$$

Because Eqs. S7 and S8 have the fitness function ϕ on the right hand side, the scaling coefficients are not necessarily be constant, but are dependent on the final size or development time through the functional form of ϕ. However, in the case of holometabolous insects, due to the assumption of energy reallocation $\phi = \phi(cL_{CG} + I_{CG})$ (Eqs. S2 and S5), we can use the following relationship:

$$\frac{\partial_L \phi}{\partial_I \phi} = \frac{\partial_L A_{EC}}{\partial_I A_{EC}} = c \quad \text{[S9]}$$

Substituting this into Eqs. S7 and S8, we obtain:

$$\frac{L_{CS}}{L_{CG}} = [1 - b_L(1-c)]^{\frac{1}{k}} \quad \text{[S7']}$$

$$\frac{t_{CS}}{t_{CG}} \approx \frac{b_L}{b_L + (1-b_I)\left\{[1-b_L(1-c)]^{\frac{k-1}{k}} - 1\right\}} \quad \text{[S8']}$$

Eventually, the scaling pattern (e.g., linear or nonlinear) depends only on four growth parameters, which appear on the right hand side of these equations: $b_I, b_L, c,$ and k. If these four parameters are constant between species, the scaling pattern is linear and the final size is proportional to the critical size. At this time, by assuming the exponential growth during the TGP (Nijhout et al., 2006; Shingleton et al., 2008; Vollmer et al., 2016), an inversely proportional relationship between the TGP and growth rate emerges:

$$\begin{cases} \text{[Final size]} \propto \text{[Critical size]} \\ \text{[Final size]} = \text{[Critical size]} * \exp(\text{[Growth rate]} * \text{[TGP]}) \end{cases}$$

$$\Rightarrow \log \frac{\text{[Final size]}}{\text{[Critical size]}} = \text{[Growth rate]} * \text{[TGP]} = \text{const.}$$

$$\Rightarrow \text{[Growth rate]} \propto \text{[TGP]}^{-1}$$

Estimation of growth parameters from experimentally measurable quantities

How can we estimate values of the growth parameters b_I, b_L, c and k in real organisms? Regarding b_I and b_L, we can use the following relationships derived from Eq. S4B:

$$b_I = \frac{\Delta I_{PRE}}{\Delta L_{PRE} + \Delta I_{PRE}} \quad \text{[S10A]}$$

$$b_L = \frac{\Delta L_{TGP}}{\Delta L_{TGP} + \Delta I_{TGP}} \quad \text{[S10B]}$$

where $\Delta X_{TGP} \coloneqq X_{CG} - X_{CS}$ and $\Delta X_{PRE} \coloneqq X_{CS} - X_0$ indicates the amount of growth in variable X (L or I) before and after the critical size attainment, respectively. Because imaginal tissues are negligible compared to larval tissues during the whole larval stage, we can assume $L(t) \gg$

$I(t)$ for $0 \leq t \leq t_{CG}$, and therefore we can expect that $b_L \approx 1$ and $b_I \approx 0$ hold true in real organisms. This means that the ratio of controllable energy is small and most energy is allocated to the larval tissues (see Figure 2 and Eq. S1). Applying this assumption to Eqs. S7' and S8', we obtain the simplified form of scaling relationships:

$$L_{CS} \approx c^{\frac{1}{k}} L_{CG} \qquad \text{[S7'']}$$

$$t_{CS} \approx c^{\frac{1-k}{k}} t_{CG} \qquad \text{[S8'']}$$

Interestingly, these equations indicate that, even in an extreme case where the controllable energy is very small, both the optimal critical size and optimal critical time do not take a limit value such as L_{CG} and t_{CG}, but rather take an intermediate value.

Under the above assumptions ($L_0 \approx 0, b_L \approx 1, b_I \approx 0$ and $L_{CG} \gg I_{CG}$), the remaining two parameters c and k can be estimated by using Eq. S2 and Eq. S4A (i.e., $L(t) \approx [g(1-k)t]^{\frac{1}{1-k}}$), respectively:

$$c \approx \frac{A_{EC}}{L_{CG}} \qquad \text{[S11A]}$$

$$k \approx 1 - \left(\frac{d \log L}{d \log t}\right)^{-1} \qquad \text{[S11B]}$$

where $d \log L / d \log t$ is the slope in the double logarithmic plot of the growth curve from hatching until growth cessation. Therefore, the value of c can be obtained from Figure 3B and the value of k can be obtained by fitting the growth curve with a power function $y = ax^b$ and then converting by $k = 1 - 1/b$ (Figure S1D).

The constancy of c between two species means that the % weight loss during the non-feeding period (i.e., the wandering larval and pupal period) is the same for those species. In contrast, the constancy of k between two species means that the two larval growth curves perfectly overlap if normalized by the final size and the total development time.

Rotation-free segmented linear regression

In a conventional segmented linear regression (SLR) (Muggeo, 2008), it is difficult to detect the breakpoint close to the end points of data for the x-coordinates. These type of data are often observed in a relationship between the larval size and the time to pupariation in *Drosophila* (Callier et al., 2013; Ghosh et al., 2013; Testa et al., 2013). The difficulty in obtaining the correct regression line is likely

due to error amplification caused by the steep slope (Figure S4A, left panel). In previous studies, as a practical approach to solve this problem, the whole data is rotated 0.5 degrees around the origin before regression (Callier et al., 2013; Ghosh et al., 2013; Testa et al., 2013). However, in this rotation-based SLR method, the rotation angle is arbitrary and must be determined optimally depending on the characteristics of existing data (such as the ratio of the data range of x to that of y). To avoid these difficulties, we developed a rotation-free SLR method that can detect a breakpoint independently of data characteristics. Because our method accounts for errors not only in y-axis (dependent variables), but also in x-axis (independent variables), error amplification does not occur no matter how steep the true slope is. Furthermore, to make the regression invariant to the scale of variables (i.e., dimension or unit), the residuals, which are defined by perpendicular distances to the regression line from the data points, are calculated after data standardization (Figure S4A, right panel). We describe the detailed procedure in the following five steps:

---

1. Convert data (X_i, Y_i) $(i = 1,2,\cdots n)$ to standardized data (x_i, y_i) according to the following formula:

$$x_i := \frac{X_i - \mu_X}{\sigma_X}, y_i := \frac{Y_i - \mu_Y}{\sigma_Y}$$

$$\mu_X := \frac{1}{n}\sum_{i=1}^{n} X_i, \mu_Y := \frac{1}{n}\sum_{i=1}^{n} Y_i, \sigma_X^2 := \frac{1}{n}\sum_{i=1}^{n}(X_i - \mu_X)^2, \sigma_Y^2 := \frac{1}{n}\sum_{i=1}^{n}(Y_i - \mu_Y)^2$$

2. In a standardized space, define a segmented linear regression equation with two segments:

$$y = \begin{cases} a_1(x - x_0) + y_0 & x \le x_0 \\ a_2(x - x_0) + y_0 & x > x_0 \end{cases}$$

3. For this regression line, define the residual sum of squares E based on the shortest distance d from each data point (i.e., the shorter one of the two perpendiculars):

$$E(a_1, a_2, x_0, y_0) := \sum_{i=1}^{n} d(x_i, y_i | a_1, a_2, x_0, y_0)^2$$

$$d(x_i, y_i | a_1, a_2, x_0, y_0) := \min\left\{\frac{|(y_i - y_0) - a_1(x_i - x_0)|}{\sqrt{1 + a_1^2}}, \frac{|(y_i - y_0) - a_2(x_i - x_0)|}{\sqrt{1 + a_2^2}}\right\}$$

4. Obtain a regression line that minimizes the residual sum of squares by the simulated annealing:

4-1. Search the new point candidate $\{a_1', a_2', x_0', y_0'\}$ by perturbing the current point $\{a_1, a_2, x_0, y_0\}$. Note that candidate points cannot be efficiently sampled unless a probability distribution in angular space is given for variations in the slopes of the straight line, a_1, a_2.

4-2. Calculate the difference of the residual sum of squares, $\Delta E \coloneqq E(a_1', a_2', x_0', y_0') - E(a_1, a_2, x_0, y_0)$.

4-3. If $\Delta E < 0$, adopt the candidate with probability 1; if $\Delta E \geq 0$, adopt with probability $e^{-\beta \Delta E}$. β is an inverse temperature parameter and takes an annealing schedule of $\beta = (1 - T/T_{\max})^{-1}$, where T and $T_{\max}$ are the current and maximum time steps, respectively, where T is initially set as 0.

4-4. Increase T by 1. If $T < T_{\max}$, return to 4-1. If $T = T_{\max}$, end the procedure and go to the next step.

5. Return the regression line to the original scale according to the following equation:

$$Y = \begin{cases} \mu_Y + \sigma_Y \left[a_1 \left(\dfrac{X - \mu_X}{\sigma_X} - x_0 \right) + y_0 \right] & X \leq \mu_X + \sigma_X x_0 \\ \mu_Y + \sigma_Y \left[a_2 \left(\dfrac{X - \mu_X}{\sigma_X} - x_0 \right) + y_0 \right] & X > \mu_X + \sigma_X x_0 \end{cases}$$

Here, the x-coordinates of the breakpoint, $\mu_X + \sigma_X x_0$, corresponds to the critical size.

To test the validity of the proposed method using the published data from the previous study on critical size (Koyama et al., 2014), we compared the breakpoint obtained by the rotation-based SLR with that obtained by our rotation-free SLR. As shown in Figure S5B, a strong correlation ($r = 0.98$) was observed between the two methods, confirming that our method is accurate.

Differences between *Drosophila* and *Manduca*

In the nine *Drosophila* species in this study, we found no large difference in the estimated threshold size values between methods A and B (Figure S3). However, in *Manduca sexta*, the value estimated by method A was usually much smaller than that of method B (Nijhout, 1975; NIJHOUT and WILLIAMS, 1974). The former is called the minimum viable weight (MVW), which can be clearly distinguished from the latter, the critical weight (CW) (Callier and Nijhout, 2013). Interestingly, when we calculate the switching point of optimal energy allocation for *Manduca* based on published literature, its value is much closer to the MVW than the CW (Hironaka and Morishita, 2017) [Table S1]. Additionally, consistent with the expectation from the optimal energy allocation model that imaginal discs begin to grow rapidly after the energy allocation switch (Figure 1B), initiation of *Manduca* imaginal disc growth (observed at 1-2 days of final instar) is slightly earlier than the attainment of the CW (usually occurs at 2-3 days of final instar) (Nijhout et al., 2006; Truman et al.,

2006). These two facts suggest that the energy allocation switch in *Manduca* occurs at the MVW rather than the CW.

In *Manduca*, it is reported that the CW can be predicted by extrapolating the log-linear relationship between larval size at each instar molt (called "Dyar's rule") (Nijhout et al., 2006). Namely, the predicted weight at a presumptive fifth to sixth instar molt is very close to the CW (note that the fifth instar is usually the final instar for *Manduca*). However, in these nine *Drosophila* species, this method of weight estimation fails to predict the CW and has an obviously larger value than the CW (Figure S5A). These observations also imply that the CW of *Manduca* refers to a physiologically different checkpoint than the CW of *Drosophila*.

A previous study suggested that the different CW behavior between *Manduca* and *Drosophila* is derived from a different JH effect in metamorphosis (Hatem et al., 2015). Consistent with this idea, topical JH application did not suppress pupation of nine *Drosophila* species, unlike *Manduca*, although the eclosion of pupae was completely suppressed for all species (Figure S5B).

References

Ashburner, M., Golic, K.G., Hawley, R.S., 2005. Drosophila : a laboratory handbook. Cold Spring Harbor Laboratory Press.

Bonduriansky, R., Day, T., 2003. THE EVOLUTION OF STATIC ALLOMETRY IN SEXUALLY SELECTED TRAITS. Evolution (N. Y). 57, 2450–2458. doi:10.1111/j.0014-3820.2003.tb01490.x

Callier, V., Nijhout, H., 2013. Body size determination in insects: a review and synthesis of size-and brain-dependent and independent mechanisms. Biol. Rev.

Callier, V., Shingleton, A.W., Brent, C.S., Ghosh, S.M., Kim, J., Harrison, J.F., 2013. The role of reduced oxygen in the developmental physiology of growth and metamorphosis initiation in Drosophila melanogaster. J. Exp. Biol. 216, 4334–40. doi:10.1242/jeb.093120

Day, T., Taylor, P.D., 1997. Von Bertalanffy's Growth Equation Should Not Be Used to Model Age and Size at Maturity. Am. Nat. 149, 381–393.

De Moed, G.H., Kruitwagen, C.L.J.J., De Jong, G., Scharloo, W., 1999. Critical weight for the induction of pupariation in Drosophila melanogaster: genetic and environmental variation. J. Evol. Biol. 12, 852–858. doi:10.1046/j.1420-9101.1999.00103.x

Ghosh, S.M., Testa, N.D., Shingleton, A.W., 2013. Temperature-size rule is mediated by thermal plasticity of critical size in Drosophila melanogaster. Proc. Biol. Sci. 280, 20130174. doi:10.1098/rspb.2013.0174

Hatem, N.E., Wang, Z., Nave, K.B., Koyama, T., Suzuki, Y., 2015. The role of juvenile hormone and insulin/TOR signaling in the growth of Manduca sexta. BMC Biol. 13, 44. doi:10.1186/s12915-015-0155-z

Hironaka, K., Morishita, Y., 2017. Adaptive significance of critical weight for metamorphosis in holometabolous insects. J. Theor. Biol. doi:10.1016/j.jtbi.2017.01.014

Hou, C., Zuo, W., Moses, M.E., Woodruff, W.H., Brown, J.H., West, G.B., 2008. Energy uptake and allocation during ontogeny. Science 322, 736–9. doi:10.1126/science.1162302

Kirk, D.E., 2012. Optimal Control Theory: An Introduction. Dover Publications, New York.

Koyama, T., Rodrigues, M.A., Athanasiadis, A., Shingleton, A.W., Mirth, C.K., 2014. Nutritional control of body size through FoxO-Ultraspiracle mediated ecdysone biosynthesis. Elife 3, e03091. doi:10.7554/eLife.03091

Lavrynenko, O., Rodenfels, J., Carvalho, M., Dye, N.A., Lafont, R., Eaton, S., Shevchenko, A., 2015. The Ecdysteroidome of Drosophila: influence of diet and development. Development 142, 3758–68. doi:10.1242/dev.124982

Mirth, C., Truman, J.W., Riddiford, L.M., 2005. The role of the prothoracic gland in determining critical weight for metamorphosis in Drosophila melanogaster. Curr. Biol. 15, 1796–807. doi:10.1016/j.cub.2005.09.017

Muggeo, V.M.R., 2008. Segmented: an R package to fit regression models with broken-line relationships. R news 8, 20–25.

Nijhout, H., 1975. A threshold size for metamorphosis in the tobacco hornworm, Manduca sexta (L.). Biol. Bull.

Nijhout, H.F., Davidowitz, G., Roff, D.A., 2006. A quantitative analysis of the mechanism that controls body size in Manduca sexta. J. Biol. 5, 16. doi:10.1186/jbiol43

NIJHOUT, H.F., WILLIAMS, C.M., 1974. Control of Moulting and Metamorphosis in the Tobacco Hornworm, Manduca Sexta (L.): Growth of the Last-Instar Larva and the Decision to Pupate. J. Exp. Biol. 61.

Ohhara, Y., Kobayashi, S., Yamanaka, N., Kaynig, V., Longair, M., Pietzsch, T., 2017. Nutrient-Dependent Endocycling in Steroidogenic Tissue Dictates Timing of Metamorphosis in Drosophila melanogaster. PLOS Genet. 13, e1006583. doi:10.1371/journal.pgen.1006583

Roff, D., 2002. Life history evolution. Sinauer Associates, Massachusetts.

Shingleton, A.W., Mirth, C.K., Bates, P.W., 2008. Developmental model of static allometry in holometabolous insects. Proc. Biol. Sci. 275, 1875–85. doi:10.1098/rspb.2008.0227

Stieper, B.C., Kupershtok, M., Driscoll, M. V, Shingleton, A.W., 2008. Imaginal discs regulate developmental timing in Drosophila melanogaster. Dev. Biol. 321, 18–26. doi:10.1016/j.ydbio.2008.05.556

Testa, N.D., Ghosh, S.M., Shingleton, A.W., 2013. Sex-specific weight loss mediates sexual size dimorphism in Drosophila melanogaster. PLoS One 8, e58936. doi:10.1371/journal.pone.0058936

Truman, J.W., Hiruma, K., Allee, J.P., Macwhinnie, S.G.B., Champlin, D.T., Riddiford, L.M., 2006. Juvenile hormone is required to couple imaginal disc formation with nutrition in insects. Science 312, 1385–8. doi:10.1126/science.1123652

Vollmer, J., Iber, D., Ahuja, C., Eswaran, H., Nijhout, H.F., 2016. An Unbiased Analysis of Candidate Mechanisms for the Regulation of Drosophila Wing Disc Growth. Sci. Rep. 6, 39228. doi:10.1038/srep39228
